# Public Awareness regarding Common Eye Diseases among Saudi Adults in Riyadh City: A Quantitative Study

**DOI:** 10.1155/2017/9080791

**Published:** 2017-07-02

**Authors:** Waleed A. Al Rashed, Amro K. Bin Abdulrahman, Ahmed A. Zarban, Mohammed S. Almasri, Abdulrahman S. Mirza, Rajiv Khandekar

**Affiliations:** ^1^College of Medicine, Al Imam Mohammad Ibn Saud Islamic University, Riyadh, Saudi Arabia; ^2^King Khalid Eye Specialist Hospital, Riyadh, Saudi Arabia

## Abstract

**Aim:**

The current study aimed to evaluate the knowledge of eye disease and awareness of eye care among the Saudi adults and to explore existing eye-related misconceptions in the community.

**Methods:**

A cross-sectional study was conducted in Riyadh city during May and June 2016. A self-administered anonymous online questionnaire was used to explore the most common misconceptions related to eye diseases and eye care.

**Results:**

Out of 1000 individuals, only 711 (71.1%) participant responses were received. The participants' acceptable knowledge (score ≥50%) was high about the eye problem in diabetes (88.6%), ocular trauma (81.2%), and other general eye diseases (91.3%), whereas low about refractive errors (63%), pediatric eye problems (51.5%), and glaucoma (14.8%). The variation in knowledge about specific ocular morbidities was significant (*p* < 0.001). The majority of participants reported sources of information about the common eye diseases and eye care encountered from the community, internet-based resources, and social media.

**Conclusions:**

The majority of the participants had awareness about the common eye diseases, whereas low percentage of participant's awareness about specific condition of eye diseases. Public eye health awareness should be more focused on social media and the internet to be able to cover the younger individuals of the community.

## 1. Introduction

On January 1, 2016, leaders of the United Nations member countries implemented the sustainable development goal (SDG) agenda up to 2030. The third most important goals of this agenda were to ensure healthy lives and promote well-being for all at all ages [1]. Recent data of the World Health Organization (WHO) show 285 million visually impaired people of all ages globally, which represent 80% of the total health burden (WHO, 2017). A joint program of the WHO and the International Agency for the Prevention of Blindness (IAPB) launched VISION 2020: The Right to Sight in 1999 to eliminate avoidable blindness by the year 2020 [2]. Therefore, there is a need for possible implementation of eye health care promotion strategies.

Knowing the degree of populations' knowledge concerning eye health and the factors that contribute to ill eye health could serve to complement such strategies. Studies measuring knowledge of eye care and diseases have been conducted in different countries and became the foundation on which eye health promotion was planned [3–5].

Misconceptions regarding specific blinding eye diseases have been studied in Arab countries, including Saudi Arabia [6, 7]. However, the current study may produce baseline information following the adoption of sustainable development goals (SDGs). This baseline information can be useful in guiding health promotion initiatives in Saudi Arabia. Furthermore, it can serve as a reference point to evaluate the impact of these initiatives in the following years.

Blinding eye diseases are more common in elderly populations, and while getting care, many are exposed to detailed knowledge about eye ailments [8]. Therefore, generating evidence-based information from adult populations would be ideal for planning health promotion strategies targeting vulnerable populations. In addition, studies about public health awareness of a group of eye-related diseases and eye health education in Saudi Arabi are scarce. The current study was aiming to assess the magnitude and determinants of knowledge regarding the eye diseases and eye care among Saudi adults in Riyadh city and to explore existing eye-related misconceptions in the community.

## 2. Subjects and Methods

### 2.1. Study Subjects

A quantitative and cross-sectional survey-based study was conducted from May to June 2016 in Riyadh, Saudi Arabia. This study randomly selected 1000 Saudi adult people aged ranging between 20 and 60 years. The participants' information was collected with the help of a local marketing company that used the Saudi Telecom Company's database. The online questionnaire along with the objectives of the study was sent to the participants. A formal online web page consent was obtained from each participant before they filled out the questionnaire. All participants were allowed to respond at their convenience and available time, and their privacy was secured. Also, participation was entirely voluntary.

### 2.2. Data Collection Methods

Based on feedback from the pilot samples, the questionnaire was subsequently modified. The questionnaire was divided into three parts. The first part consisted of 11 items, which referred to demographic information. The second part consisted of 25 items, which measured the eye care and eye health information. The third part asked about the resources of eye health and care information.

SurveyMonkey (www.surveymonkey.com) was used to collect participants' responses [9]. All participants were immediately directed to an online page which contained the correct information about eye diseases and eye care that have been presented in the questionnaire, along with important eye health education comments prepared by a senior ophthalmologist. Repeated submissions from the same participant were prohibited through linking each response with their IP address in the SurveyMonkey web page. A Likert scale was used to measure and analyze the responses where 1 = true, 2 = false, and 3 = I do not know [10]_._ For a question, true response marked +2 score, false response marked −2 score, and I do not know response marked −1 score. The sum of the scores on all 25 questions provided the final score for each participant. The participants' knowledge can be categorized as “excellent” if they obtained more than 75% score, “good” if they obtained a score between 50 and 75%, and “poor” if they obtained less than 50% score. The participants' score ≥ 50% was considered as an acceptable level of knowledge.

### 2.3. Statistical Analysis

The data were collected from SurveyMonkey, tabulated using Microsoft Excel 2010, and analyzed using SPSS software (Version 23, IBM Corp., Armonk, NY, USA). Descriptive statistics was employed to measure the frequencies. Pearson's chi-square test and odds ratios (ORs) were used to evaluate and quantify the associations between a categorical outcome and the variables under consideration. During the entire analysis, the statistical significance level was considered as *p* ≤ 0.05.

## 3. Results

The questionnaire was distributed among the 1000 Saudi adults; 711 of them completed the questionnaire. The response rate was 71.1%. The mean (SD) age of the participants was 35.32 (12.2) years. The internal reliability of the questionnaire was high (Cronbach's alpha = 0.803). Demographic information of the participants is given in Table 1. The majority of participants were married and government employees and had university education. Most of the participants' knowledge test score about the eye health and care was good (68.2%), whereas some participants had excellent knowledge (17.3%), and very few participants had poor knowledge (14.4%). The mean (SD) age of participants with excellent knowledge was 38.4 (10.5) years, whereas with below excellent level of knowledge was 34.8 ± 12.5 years. The result showed significant association between an increased age with better knowledge level (*p* = 0.002).

The knowledge of eye health and care and its association with different variables are given in Table 2. The female participants had slightly higher knowledge compared to the male participants (OR = 0.6, *p* = 0.001). The participant's education at preuniversity level scored less compared to those having high education level (*χ*^2^ = 7.2; *p* = 0.007). Similarly, less percentage of unmarried participants have acceptable knowledge compared to the married, divorced, and widow participants (χ^2^ = 7.3, *p* = 0.0007). Also, the participants with a family member suffering from the eye diseases have significantly higher knowledge than those having no eye disease in their family (*χ*^2^ = 6.8, *p* = 0.009). However, participants' occupational level did not have a significant effect on the knowledge level.

The level of acceptable knowledge related to specific morbidities is given in Table 3. The participants' knowledge about the eye problem in diabetes, ocular trauma, and general eye diseases was high (88.6, 81.7, and 91.3%, resp.), whereas knowledge about refractive errors, pediatric eye problems, and glaucoma was low (63.0, 51.5, and 14.8%, resp.) (*χ*^2^ = 418; *p* < 0.001). Participants' sources of information about the eye diseases and eye care are shown in Table 4. The most commonly reported sources were members of the community (55.4%), internet-based search engine (45.1%), and social media (28.6%) and book and brochures (28.7%).

## 4. Discussion

To the best of our knowledge, this is the first study addressing the awareness and misconceptions of various eye diseases and eye care among Saudi adults in Riyadh city with such a large sample size, as there was no comprehensive study conducted in Riyadh city evaluating a group of different eye disease-related knowledge at once.

Disease-specific studies about glaucoma, diabetic retinopathy, strabismus, and cataract were conducted [5, 11–13]. However, most of those studies were in the elderly population and did not include younger adults. Some were conducted in eye clinics or in hospitals [11] and did not represent knowledge among the healthy population in the community.

Although female adults on average had better knowledge regarding eye diseases and eye care, however, a study in Nepal showed better knowledge among males compared to females. Higher educational levels of Saudi females could explain the contrasting observations in these two studies. Furthermore, the socioeconomic differences between the two countries might affect the level of knowledge about eye diseases and eye care. For example, Saudi female adults are more likely to have a sufficient lifestyle and an acceptable financial support by their families. This may indicate that they have less stress toward getting a living and thus may have a higher chance to come across eye health and eye disease advocacy in the internet.

Older Saudi adults had better knowledge as compared to younger ones. This was supported by a study in Nigeria that showed older participants were predictors of better knowledge about glaucoma [14]. It is known that older persons are more prone to get diseases. This may encourage older individuals to be more caring to visit their physicians in comparison to younger individuals. Also, older persons are more likely to be retired. So, they may have more free time to read or watch health education programs.

Moreover, the public health awareness, nowadays, may have been improved in Saudi Arabia as compared to the last two decades. Furthermore, most of the study population in Riyadh city had higher educational level (85.3%) as shown in Table 2. Therefore, older participants had combined both higher education level and long life experience. So, older Saudi adults were most likely had encountered many health promotion campaigns in shopping malls, public places, primary health care centers, hospitals, TV medical programs, and/or radio programs. Hence, they tend to be wiser and concerned about their health status in comparison to younger adults.

In the present study, 63% of the study sample had an acceptable level of knowledge about the eye in diabetes. The prevalence of diabetes in the Saudi adult population is 18% [15]. Hajar et al. [16] found that among the older population (50 years or older), 22.4% were suffering from diabetes. Additionally, 28% of them had diabetic retinopathy. Many adults who attend hospitals, especially diabetics or relatives of diabetics, can be exposed to campaigns for prevention of diabetes and diabetic retinopathy. This might explain why over 50% of the participants had good knowledge about the eye in diabetes. Furthermore, based on the high prevalence of diabetes shown previously among Saudi adult population, it is thought that many Saudi families had at least one member having diabetes (e.g., first, second, or third degree relatives). Therefore, it was noticed that huge efforts were done toward public health promotion about diabetes in Saudi Arabia, which was mainly supported by the Ministry of Health and other health-related sectors.

Glaucoma-related knowledge was much lower in our study. It was also noted to be 7% in Bangladesh, 15.8% in Nigeria, and 8.3% in north India [4, 17]. Prevalence of glaucoma as a cause of complete blindness is considered high, as it had reached up to 10% in our subregion of Eastern Mediterranean countries [18]. The previous result could be explained by the fact that glaucoma is more common among older individuals. This could make the younger adults (below 50 years) less likely to read about this disease. In addition, glaucoma may not be a very common disease to the community in Saudi Arabia, which was neither addressed very often in public health campaigns nor in health advocacy in social media and the internet. Hence, this result may indicate that there was a great gap of eye public awareness, especially regarding glaucoma.

Information from other community members, the internet, and social media was the leading sources from which participants obtained knowledge on eye diseases and eye care. Community members were shown to be the highest used source to gain information (55%) as shown in Table 2. This also supports the findings of having relatives with eye diseases and improvement of level of knowledge about eye diseases and eye care. The study sample may indicate that Saudi individuals were in favor of asking people around them in community for a trustworthy eye health information (e.g., friends, family members and relatives, neighbors). However, others were more comfortable with the new modern ways to get quick and up-to-date reliable information through internet search, social media applications/programs, as they are user-friendly and always accessible.

## 5. Conclusion

The majority of the participants were moderately aware about the eye diseases and common causes of eye problems. Therefore, our data suggest that there is a need for eye healthcare interventions, focus on weaker areas of knowledge, particularly on glaucoma. The knowledge of common eye diseases and awareness of eye care could lead to an increase in understanding and management of eye health, and thereby, it may reduce visual impairment and cost of eye care.

## 6. Limitations

The conventional five-point Likert scale was not used in the questionnaire as it may complicate the simplicity of questionnaire to our sample and also to avoid uncertain answers by participants. Hence, a three-point scale was used instead.

## Figures and Tables

**Table 1:** Demographic information of the Saudi adults in Riyadh city 2016.

Variables	*n* (%)
Gender	
Male	350 (49.2)
Female	361 (51.8)
Marital status	
Single	231 (32.5)
Married	457 (64.3)
Divorced	17 (2.4)
Widowed	6 (0.8)
Education level	
Preuniversity	177 (24.9)
University	422 (59.4)
Postgraduate	108 (15.2)
Illiterate	4 (0.6)
Occupation	
Student	165 (23.2)
Employed	335 (47.1)
Self-employed	7 (1.0)
Retired	46 (6.5)
Unemployed	158 (22.2)
Eye/systemic health problem	
Yes	94 (13.2)
No	617 (86.8)
Family with eye/systemic disease	
Yes	421 (59.2)
No	165 (23.2)
Do not know	125 (17.6)
Knowledge of eye care/diseases	
Excellent	102 (14.3)
Good	485 (68.2
Poor	124 (17.4)

**Table 2:** Acceptable level of knowledge about the eye care and eye diseases among Saudi adults in Riyadh city 2016.

Determinants	Participants	Acceptable level of knowledge		
		*n* (%)	95% CI	Validation
Gender				
Male	350	276 (78.9)	76.7–81.0	OR = 0.6;
Female	361	311 (86.1)	84.3–88.0	95% CI (0.4–0.9); *p* = 0.001
Education level				
Pre-university level	231	177 (76.6)	73.8–79.4	*χ* ^2^ = 7.2
University level	457	390 (85.3)	83.7–87.0	*p* = 0.007
Postgraduate level	17	15 (88.2)	80.4–96.0
Illiterate	6	5 (83.3)	68.1–98.5
Occupation				
Students	165	130 (78.8)	75.6–82.0	*χ* ^2^ = 1.2
Government employee	263	221 (84.0)	81.8–86.3	*p* = 0.3
Private employee	72	57 (79.2)	74.4–84.0
Looking for a job	36	31 (86.1)	80.3–91.9
Retired	46	38 (82.6)	77.0–88.2
Business person	7	6 (85.7)	72.5–98.9
Unemployed	122	104 (85.2)	82.0–88.5
Marital status				
Single	231	177 (76.6)	73.8–79.4	*χ* ^2^ = 7.3
Married	457	390 (85.3)	83.7–87.0	*p* = 0.007
Divorced	17	15 (88.2)	80.4–96.0
Widowed	6	5 (83.3)	68.1–98.5
Suffering from eye disease				
Yes	94	78 (83.0)	79.1–86.9	OR = 1.03;
No	617	509 (82.5)	81.0–84.0	95% CI 0.6–1.8; *p* = 0.9
Relatives with eye diseases				
Yes	421	366 (86.9)	85.3–88.6	*χ* ^2^ = 6.8
No	165	130 (78.8)	75.6–82.0	*p* = 0.009
I do not know	125	91 (72.8)	68.8–76.8
Total	711	587(82.6)	81.1–84.0	

95% CI: 95% confidence interval.

**Table 3:** Levels of ocular morbidity-related knowledge among Saudi adults in Riyadh city 2016.

Ocular morbidity	Acceptable level of knowledge	Poor level of knowledge	Validation
	*n* (%)	*n* (%)	
Refractive errors and the cornea	448 (63.0)	263 (37.0)	χ^2^ = 418 *p* < 0.001
Pediatric ophthalmology	366 (51.5)	345 (48.5)	
Glaucoma	105 (14.8)	606 (85.2)	
The eye in diabetes	630 (88.6)	81 (13.7)	
Ocular trauma	581 (81.7)	130 (18.3)	
Miscellaneous	649 (91.3)	62 (8.7)	

**Table 4:** Sources of information for accessing knowledge regarding eye diseases and eye care among Saudi adults in Riyadh city 2016.

Source of information	*n* (%)
Internet-based resources	321 (45.1)
Social media	203 (28.6)
Periodicals	95 (13.4)
Television & radio	154 (21.7)
Eye care professionals	221 (31.1)
Family physician	57 (8.0)
Pharmacist	61 (8.6)
Community	394 (55.4)
Books and brochures	204 (28.7)
Health awareness campaign	70 (9.8)
Others	68 (9.6)
